# Effectiveness of a universal classroom-based preventive intervention (PAX GBG): A research protocol for a matched-pair cluster-randomized controlled trial

**DOI:** 10.1016/j.conctc.2017.08.013

**Published:** 2017-08-30

**Authors:** Karin Streimann, Aire Trummal, Kai Klandorf, Kirsti Akkermann, Merike Sisask, Karmen Toros, Anne Selart

**Affiliations:** a National Institute for Health Development, Tallinn, Estonia; b School of Governance, Law and Society, Tallinn University, Tallinn, Estonia; c Institute of Psychology, University of Tartu, Tartu, Estonia; d Center for Cognitive Behavior Therapy, Tartu, Estonia; e Estonian-Swedish Mental Health and Suicidology Institute, Tallinn, Estonia; f Institute of Mathematics and Statistics, University of Tartu, Tartu, Estonia

**Keywords:** Mental health, Intervention, Children, Good behavior game, Prevention, Health promotion

## Abstract

**Introduction:**

The PAX Good Behavior Game (PAX GBG) is a universal classroom-based program that promotes children's mental health. In Estonia, the intervention is delivered to first grade students (aged seven to eight) within the regular school curriculum. The current work describes a protocol for a cluster-randomized controlled trial (RCT) of the PAX GBG conducted in Estonia.

**Design and methods:**

This is an ongoing, pragmatic, two-year, matched-pair, cluster-RCT conducted in Estonian elementary schools. Schools were matched to pairs based on their geographical location and number of students per classroom. One school in each pair was randomly selected to receive the intervention and the other placed on a wait-list as a control. 42 schools provided baseline data during the autumn of 2016. Data is collected at two more points in time – seven months and 19 months post-baseline. Outcomes of children's mental health and behavior are measured by the teacher- and parent-rated Strengths and Difficulties Questionnaire, parent-rated Swanson, Nolan, and Pelham – IV Questionnaire and the Go/No-Go task completed by children. Teachers also rate their self-efficacy and overall classroom behavior.

**Discussion:**

This study aims to test the effectiveness of the intervention in Estonian classrooms with a newer version of the rigorously tested GBG program. To our knowledge, this study is the first to measure the effects of the intervention on children's inhibitory control, which has been associated with externalizing problems in the literature. The results from this trial will provide further understanding into how the program influences children's well-being and self-control.

**Trial registration:**

ClinicalTrials.gov registry (NCT02865603).

## 1 Introduction

Children have the right to live and grow up in an environment that provides the best possible conditions for their health [1]. A child's mental health and well-being arise through interactions between individual, social and environmental factors that shape their behavior and choices [2]. Psychological well-being, personal and social competence, strong attachment to parents and a supportive school environment are all powerful factors that contribute to children being less vulnerable to drug use, mental health problems, dropping out of school, delinquency, violence and risky sexual behavior [3].

School is a frequently used setting for preventive interventions targeting children [4] as they spend large amounts of their time there. Several universal school-based prevention programs have demonstrably reduced psychological and behavioral problems in children [5]. One of these is the Good Behavior Game (GBG), a behavior management strategy that has been used for more than 40 years [6]. The GBG addresses disruptive and aggressive behavior during middle childhood, important behavioral antecedents of adolescent drug use and mental health problems [7].

The goal of the GBG is to increase children's self-regulation and peer-cooperation [5]. Interventions addressing self-control during childhood can prevent a range of difficulties, for example, substance dependence and criminal offences during adulthood [8]. At the same time, cooperative social environments foster children's successful development and promote their mental health [9].

Several studies across the United States, the Netherlands and Belgium have attested the effectiveness of GBG and found that the GBG reduces behavioral and emotional problems [10], [11], [12], [13], [14], [15], [16], prevents the use of tobacco, alcohol and illicit drugs [7], [17], [18], [19] and improves educational attainment [7], [20]. Although, in general, the program has proven to be effective, one study also determined that the intervention did not have a positive impact on children from dysfunctional families or those with combinations of behavioral and social risks [21].

The PAX version of the GBG (PAX GBG, developed by the Paxis Institute in the United States) was implemented in Estonia for the first time in 2014 by the Estonian National Institute for Health Development (NIHD). In addition to the original classroom-based game where students are reinforced for their mutual success in withholding inappropriate behavior, PAX GBG includes evidence-based kernels or behavior influence strategies [22]. There are four categories of kernels based on their effect: antecedent, relational, physiological and reinforcement. These kernels help children prepare for and achieve goals, reduce anxiety and offer rewards when effective behavior has occurred [23]. More recent studies conducted in the United States have shown that the PAX version of the game reduces hyperactivity, increases prosocial behavior, improves children's academic skills and promotes teacher's self-efficacy [24], [25], [26].

The aim of this paper is to present the research protocol for the study designed to evaluate the impact of PAX GBG on students' mental health and behavior as well as teacher's self-efficacy in Estonia compared with the waiting list control condition. The newer version of the intervention (PAX GBG) has only been experimentally evaluated in English-language environments. Amendments were made on surface level (adapting language and images) and the underlying logic of the program was preserved during the adaptation in Estonia. At the same time, the adaptation to a new country and language, as along with the novel socioeconomic, educational and cultural environment, could feature prominently with regards to the effectiveness of the intervention [27].

## 2 Methods/design

### 2.1 Design

**Fig. 1:**
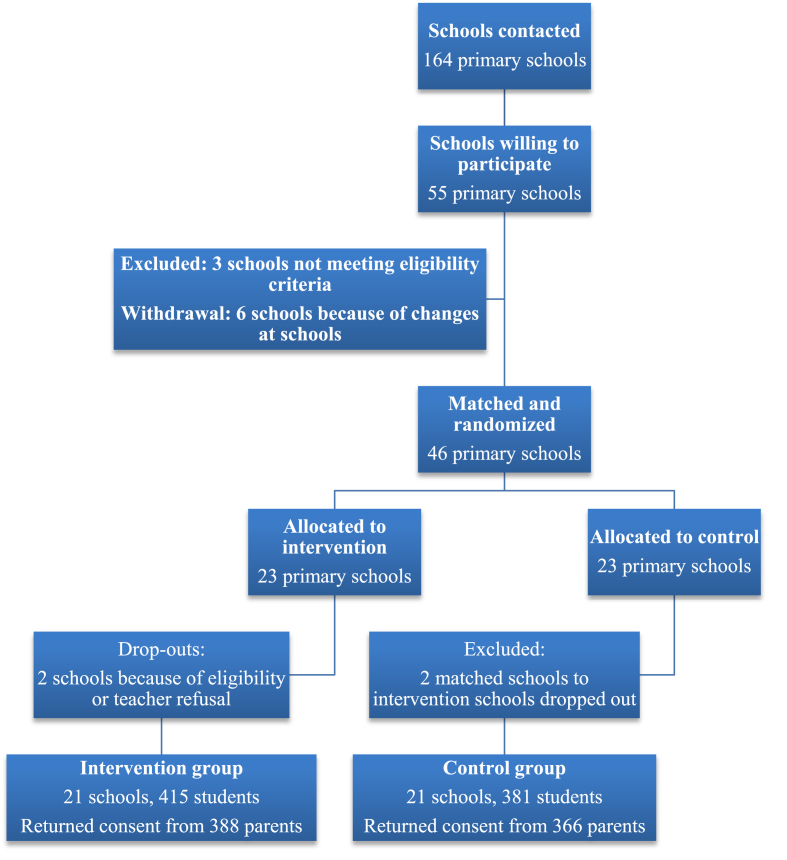
Participant flow.

This study is an ongoing pragmatic, two-year, cluster-randomized waitlist-controlled trial conducted during school years, 2016/17 and 2017/18, in Estonia. One first grade class from each school among 42 schools participates in the study. Fig. 1 outlines the participant flow.

### 2.2 Study setting

The study setting is Estonian mainstream primary schools *(n*=*42).*

#### 2.2.1 Inclusion criteria

Schools were suitable to participate if they had:

- the Estonian language as the instruction language;
- at least 13 pupils in the first grade class of the 2016/17 school year. A minimum cluster size was set to ensure enough pupils took part in the study and for better data monitoring;
- a first grade teacher who volunteered to participate in the study over the course of its full period of two years.

#### 2.2.2 Exclusion criteria

Schools were not suitable to participate if they:

- were focused solely on children with special educational needs as the intervention has not been tested in schools for students with special needs in Estonia;
- had the Russian language as the instruction language - the intervention has not been piloted yet with pupils from Russian-language classrooms;
- had single-sex classrooms in first grade in order to balance gender within both arms of the trial;
- implemented the ‘Kiusaamista Vastaan’ (KiVa) program, in order to reduce the risk of other evidence-based prevention programs affecting the results. KiVa is a bullying prevention program developed in Finland [28], that uses a whole school approach. As its components aim to affect skills, behavior, and classroom and school climate, it is assumed that it has a positive impact on children's social and emotional well-being, a primary outcome in this study.
- implemented the PAX GBG (in one or several classes) so as to reduce the risk of contamination.

#### 2.2.3 Participants

Participants are students from participating schools. In the 2016/2017 academic year the participants are first grade students (seven to eight years old) and in 2017/18 participants are in second grade (eight to nine years old).

### 2.3 Recruitment

A list of all Estonian-language primary schools was obtained from the education database, EHIS, administered by the Ministry of Education in the spring of 2016. Schools that had at least 10 first grade students per class in 2015/16 were selected to be contacted as the class sizes in 2016/17 were not known yet.

Invitations to take part in the study were sent in the spring of 2016 to 164 schools, which fit with the inclusion criteria. Invitation forms included a brief introduction of the program, the conditions of participation and a description of the goal and procedures of the study. If a school was interested in participating, the representative from the school signed the request form and wrote the name of the volunteering first grade teacher. By signing the form, the school conveyed that they understand the conditions for participation (e.g., the random assignment of schools to the intervention and control arms of the trial, willingness not to sign up for the KiVa program during the study period, agreement to participate in the intervention-related activities).

The incentives for schools to participate in the study were PAX GBG materials, participation in a three-day PAX GBG training and mentoring support for the teacher for one year.

55 schools sent back the interest form. Six of them withdrew their participation by the summer. Reasons for withdrawal were changes with personnel and lack of time. Three schools did not meet the eligibility criteria (not enough students or Russian as an instruction language) and were excluded, leaving 46 schools for the trial. All withdrawals and exclusions happened before randomization.

### 2.4 Randomization

46 schools were matched to pairs by geographical location and class size. Matching was used to facilitate implementation all over Estonia. There are 15 regions in Estonia, 11 have a local trained PAX GBG mentor. To ensure regional coverage of schools implementing the intervention (which is a regulation set by the funder) and to provide mentors with work within reasonable distance, matching was decided as the best option for achieving the research- and implementation-related goals.

Blocking or stratification is usually used in randomized experiments to improve precision [29]. In addition to accomplishing implementation-related goals, pairing (two schools per block) is expected to reduce intervention and control group differences at baseline and increase the statistical power. However, matched randomization also has several weaknesses [30] that will be taken into account within the study (i.e. if a school exits the trial after enrollment, the paired school will also be dropped; analytical limitations).

After matching, pairs were randomized into two groups. One school from each pair was randomly selected to receive the PAX GBG or to continue their activities without receiving the intervention (business as usual/BAU). The intervention is targeted at Year 1 students and hence one of the 1st classes was selected randomly from each of the participating schools (if there was more than one 1st grade).

Cluster randomization was conducted in September 2016 by an independent statistician from the Estonian-Swedish Mental Health and Suicidology Institute (ERSI). A random number generator, which employs atmospheric noise to generate truly random numbers, was utilized [31].

Following randomization and baseline data collection, the schools were contacted by phone and the results of the randomization were explained. Two schools from the intervention group dropped out after the randomization and hence their matched pairs in the control group were also removed from the study. One school wanted the third grade to participate (instead of the first) and the other was not able to ensure it was possible for the teacher to participate in the PAX GBG trainings.

### 2.5 Minimum detectable effect size

A calculation was carried out to estimate the effect size the trial could detect as statistically significant. Cohen's d was used as an effect size measure. As an outcome variable, the *Teacher-Rated Strengths and Difficulties Questionnaire* (SDQ) total difficulties score at first post-assessment was considered.

For calculating the minimum detectable effect size (MDES) we considered two scenarios. For the first, we took into account the matched-pair design. However, as our matching criteria had presumably only weak associations with the outcome variable, the MDES calculations were also performed using simple cluster random assignment. In both cases, we used a significance level of 0.05 and a power value 0.8; average class size was an estimated 20 children. For the first scenario an intra-cluster correlation coefficient (ICC) of 0.15 was assumed for children in the same pair of schools. The effects of the pairs of schools were treated as random - with random intercepts and random effects on intervention. To account for intervention effect heterogeneity values of 0.5 and 0.75 were employed as a ratio of the variance of the intervention effect between pairs to the variance between pairs. On these conditions, 21 pairs with one intervention and one control school resulted in a MDES of 0.257 (or 0.286 if the random effects variance ratio was 0.75). Adding covariates to the children level (i.e. gender and baseline score) with an assumption of 20% variance reduction decreased the MDES value to 0.243 or 0.273.

With the second scenario, we discarded the level of pairs and assumed an ICC of 0.15 for children in the same school. The ICC for the primary outcome measure was based on earlier research in the field [32], [33]. The effects of the schools were considered as random intercepts. With 42 schools randomized to the intervention or control groups on a 1:1 bases, this scenario resulted in a MDES equal to 0.389. Again, adding children-level covariates (assuming a 20% variance reduction) diminished the MDES, yielding 0.380.

In other school-based intervention trials, the effects on students' mental health and well-being were found to vary between small and moderate [34]. Previous trials of GBG have found the effects on students behavior also to vary between small and moderate [35], [36]. Based on the calculations, we are aware that statistical power is an issue with this trial and we might be able to detect moderate effects.

### 2.6 Human subject protections

Ethical approval for the study was received from Tallinn Medical Research Ethics Committee on 16 of June 2016 (Decision No. 1487).

#### 2.6.1 Active consent

Study participants were informed and consent was acquired in the following way:

1. An administration representative from each school filled out and signed an informed consent form to apply to take part in the study. By signing the form, the school assured that they understood the conditions for participation (criteria for inclusion and exclusion of schools are described in Section 2.2).
2. All parents of the children studying in the 42 classes participating in the trial were notified about the study in September 2016 and had three weeks to decide for their children to be included in the study and take part in the intervention. Parents received mail from their child's teacher that included the information letter and consent form. The information letter introduced the PAX GBG, aims and randomization principles of the study and data collection methods (e.g., children completing Go/No-Go tasks, teachers and parents evaluating children). Confidentiality issues were addressed, and it was emphasized that taking part in the study was voluntary, data would be collected on children whose parents agreed to the process and parents could elect to quit participating in the study at any time. In addition, a link to a short video [37] describing the study was added.
Parents had to choose if they agreed or not with taking part in the study. It was all-encompassing opt-in consent; consenting parents approved data collections from children, teachers and themselves all at once. Parents who agreed had to select whether they wanted to fill in questionnaires on paper or online. Parents could return a hard copy of the signed consent form to the school or send a digitally signed form to the NIHD via email. Parents also had the option of requesting a consent form in Russian.
3. Each child's verbal consent is obtained before each time one completes the Go/No-Go task. Experimental procedures are explained to the child prior to the task and children are informed about their right to stop completing the task at any time. Only children whose parents agreed to take part in the study complete the Go/No-Go task.

#### 2.6.2 Data management during the research

Each student whose parents conceded to participate was assigned a study code, which protects confidentiality and allows for linking the data collected at different time points. Parental questionnaires include the study code and do not contain any information which would permit distinguishing individuals.

Teachers' questionnaires include the study code as well as the name of the student. The student's name is added on the teacher's questionnaire so that the teachers would know about whom they are filling out the questionnaire (as they need to fill out one questionnaire per each student in their class). The teachers' questionnaires are inserted to the database only with the study code. Same teacher stays with the class from first until fourth (included) grade in Estonia; hence teacher change between data collection points is not likely.

Only the research team members can access locked data containing children's names together with their codes. All databases hold only non-identifiable data and are accessible exclusively to the research team. The data will be analyzed anonymously and results presented in generalized format.

### 2.7 Intervention

As described earlier, the PAX GBG includes a classroom-based game and evidence-based kernels. Teachers implement the methodology daily within the regular school curriculum, using kernels during all lessons where possible and GBG every day starting from the training. All children from the intervention classes receive the intervention regardless of whether they participate in the study or not.

The PAX GBG begins with creating a shared class vision displayed in the classroom. For developing and maintaining a supportive classroom environment evidence-based kernels are utilized daily. For example PAX Quiet (a cue for attention), PAX Voices (a cue for expected voice levels), Timer (to improve focus and increase time engaged in learning), PAX Stix (to increase equality and participation), Granny's Wacky prizes (motivators to increase wanted behaviors), OK/Not OK desk cards (providing feedback to groups and individuals), and PAX Tootle notes (express appreciation, thanks and recognition of accomplishments).

Once the kernels are used regularly in the classroom, the GBG is introduced and played. To play the game, the class is divided into teams. Teams work cooperatively and if they do not exceed a specified criterion of previously defined inappropriate behaviors within the game period, they win a simple activity reward (selected from Granny's Wacky prizes). At the beginning, children play the game for brief periods, with game sessions lengthening over time [22].

Competing as groups allows rewarding positive rather than deviant behavior. The students that are grouped together influence and assist each other to win the game, leading to the internalization of norms for appropriate behaviors in school and other settings [38]. Group rewards are conditional on the behavior of a group's members and, as such, supports cooperative behavior in the classroom [39]. Potentially destructive aspects of between-group competition are managed by periodically shuffling the composition of the groups [39].

#### 2.7.1 Implementation of the intervention in Estonia

PAX GBG was adapted to Estonian schools during the school year of 2014/15. In the previous two years, 41 classes participated in the program.

There are 22 active mentors (currently coaching teachers) in Estonia. The mentors all passed a three-day training conducted by the PAX GBG program developer, Dennis Embry, as well as by PAX GBG lead international trainer, Claire Richardson, and took part in continuing education training organized by the NIHD. The Estonian PAX GBG mentors conduct teacher trainings in Estonia, lasting three days altogether. Each training day was carried out separately (i.e., not in sequence). Compared with United States, where teachers normally take part of 1-day training and receive one additional booster session [24], the training period in Estonia was extended in 2015 to improve implementation fidelity.

The first training day for intervention schools' teachers was conducted at the end of October 2016 and focused on theoretical background of PAX GBG. How to increase a child's well-being, how to use kernels in classroom and the scientific background of the PAX GBG was discussed. Teachers also received PAX GBG materials (manual, posters, harmonica, desk cards, Tootle Note blanks, PAX Stix, timer, and Granny Wacky Prizes cards). The second training day was two weeks later in November 2016. Before that, teachers had to finish home assignment, which included familiarizing themselves with the teacher's manual, creating a shared vision with children and practicing kernels in their classroom. During the second day, the teachers shared their first experiences and learned the classroom-based game.

An additional one-day training took place in February 2017. Topics like assigning roles to students, playing the game without notifying the students and using PAX GBG outside the classroom were discussed. The third day of training was open for all practicing teachers to take part in regardless of which year they commenced administering PAX GBG in the classroom.

#### 2.7.2 Implementation support system in Estonia

PAX GBG mentors support implementation of the intervention and regularly visit the classroom over a one-year period. Per each implementing teacher, the mentor spends approximately 30 h per school year on class visits that include coaching sessions. During the 2016/17 school year, mentors visit each class between 10 and 14 times. They also provide counseling via e-mail and phone. In the beginning, the visits occur every two to three weeks. When a teacher feels more confident, the visits are less frequent. During the second year (in this case, 2017/18), teachers can continue using the PAX GBG methods and participate in the third day of the training in the winter of 2018. They also receive up to two visits from the mentor at the beginning of the school year to encourage continuing with the intervention. There is no cut-off for the intervention and the implementation of the methodology is expected to become a daily practice for teachers, even as they do not receive regular support during second school-year from mentors.

One school visit normally lasts two to 3 h. During the visit, the mentor observes the classroom environment and the use of PAX GBG. After the observation, the mentor makes recommendations for the teacher. During each school visit, mentors fill out observation forms (implementation rubric and progress evaluation forms), which supplies mentors with input pertaining to the progress and implementation quality of the intervention in the classroom.

### 2.8 Controls

The trial uses a waitlist control design and PAX GBG will be implemented in the control schools after the end of the trial, if schools are still interested to use PAX GBG by that time. As such, control schools carry out classroom work as usual.

To monitor what control schools deliver in the classrooms, teachers provide information about the methodology and programs they use to support children's wellbeing during baseline data collection.

### 2.9 Objectives and outcome measures

The study's main objective is to evaluate whether PAX GBG affects children's overall mental health (primary outcome), as measured by the total difficulties score on the teacher-completed Strengths and Difficulties Questionnaire (SDQ) [40].

Secondary objectives are to examine whether intervention affects:

- aspects of children's social and emotional well-being, as measured by the subscales of SDQ, completed by teachers and parents (see below for further details);
- children's inhibitory control, as measured by the visual computerized Go/No-Go task completed by children;
- children's symptoms of attention deficit hyperactivity disorder (ADHD), as measured by the parent-completed Swanson, Nolan, and Pelham – IV Questionnaire [41];
- teacher's self-rated efficacy in classroom management, as measured by the teacher-completed Teachers' Sense of Efficacy Scale [42]; and
- classroom's overall behavior, as measured by the teacher-completed four-item measure [43].

Additional data is also collected about intervention fidelity.

#### 2.9.1 Primary outcome measure

The Estonian version of the SDQ with an impact supplement is employed to assess children's mental health [40]. SDQ is a brief behavioral screening questionnaire for assessing four-to 17-year olds, which has been applied internationally and demonstrated satisfactory validity and reliability across different populations [44]. The SDQ consists of 25 statements rated on a three-point Likert scale from (0) not true to (2) certainly true. It comprises five subscales - emotional symptoms, conduct problems, hyperactivity/inattention, peer relationship problems and prosocial behavior. Responses to the subscales, apart from the prosocial subscale, lead to a total difficulties score, which has been found to be a psychometrically sound measure of overall child mental health problems [45], [46]. Ratings of child distress and the impact of difficulties combine to form the impact supplement scale. In addition to using SDQ scores as continuous variables, scores will be classified to identify likely cases with mental health disorders. Due to the lack of normative data on this measure in Estonia, cutoff scores from the United Kingdom will be used instead [47].

Teacher SDQs (primary measure) are compared with a single-sided version (without the impact supplement) of the parent-rated SDQs [48]. Data triangulation is used to increase the credibility and validity of the results [49].

#### 2.9.2 Secondary outcome measures

The *visual computerized Go/No-Go task* is used to assess children's inhibitory control. The PAX GBG seeks to improve children's self-regulation skills through rewarding inhibition of aggressive, disruptive, impulsive and inattentive behavior [7]. Response inhibition serves as a cognitive marker for the study of impulsivity, while its manifestation in childhood is associated with delinquency, antisocial behavior, ADHD and drug addiction [50]. Go/No-Go task has demonstrated to be the most sensitive neuropsychological measure of impulsivity that reflects changes in response inhibition in real time [51].

The Go/No-Go task was developed for the purposes of the present study specifically for children between the ages of seven and eight. The task was developed during the summer of 2016 by the Center for Cognitive Behavior Therapy (in collaboration with the private limited company, Science Programming Team) and piloted with 52 children between seven- and eight-years old. The task is set up with the Java program and the number of commission and omission errors together with reaction times for both go and no-go tasks are registered.

12 pairs (24 schools) were randomly selected by an independent statistician from ERSI to take part in the assessments. As conducting assessments at each school takes one to two days, and time for the data collection is limited (three weeks during each wave), half of the schools taking part in the study were randomly chosen to complete the Go/No-Go tasks.

The primary purpose in the Go/No-Go task is to inhibit prepotent responses. The stimuli - pictures of animals - are presented in the center of a laptop computer screen. Specifically, pictures are presented for 1000 ms with 1000 ms intervals on a 13.3 inch screen. There are three blocks in total, each block containing four series with three Go and one No-Go condition appearing in random sequence. 30% of stimuli are distractors (i.e., No-Go stimuli) and 70% targets (i.e., Go stimuli). Children are instructed to fixate on the cross in the middle of the screen and press the spacebar with their dominant hand as quickly and accurately as possible when the target stimulus is presented and to inhibit their response when a distractor stimulus appears. Reaction times are measured and the numbers of commission and omission errors are registered for each block. Reaction times reflect self-control associated to stimuli, commission errors problems in behavioral inhibition and omission errors attentional difficulties. The experimenter reads loudly the instructions featured on the computer screen before each series. The practice trial was added before the experiment to control that children understood the nature of the task. It takes between seven and 8 min per child to complete the practice trial and experiment.

The task is conducted individually in a quiet room at a child's school during regular school hours by master- or doctoral-level psychology students. All task conductors were instructed by the task developer and NIHD, written guidance on how to administer the task was also provided.

*The Swanson, Nolan, and Pelham – IV Questionnaire (SNAP-IV)* is utilized to measure the symptoms of ADHD among children. Each of the 18 items of the SNAP-IV assesses ADHD core symptoms of hyperactivity/impulsivity and inattention [41] and are rated by parents. Items are rated on a four-point scale from (0) not at all to (3) very much. The SNAP-IV questionnaire has acceptable reliability and it satisfactorily distinguishes children withdifferent levels of ADHD concerns [41]. There is no normative data collected from Estonia, hence the original parental cutoff scores originating from United States will be used [52]. The SNAP-IV was adapted to Estonian for the Estonian Children Personality Behavior and Health Study [53].

*The Teachers' Sense of Efficacy Scale (TSES)* is employed to gauge teacher's self-rated efficacy and has demonstrated to be reasonably valid and reliable measure [42]. A 24-item measure evaluates teachers' perceptions of their sense of effectiveness as teachers on three subscales - student engagement, instructional practice and classroom management. Responses are supplied on a nine-point scale for each item from (1) nothing to (9) a great deal. Mean scores are calculated for each scale with a higher score indicating a greater sense of efficacy. The cross-cultural adaptation of the instrument was conducted for NIHD in 2015 by The Center for Applied Social Sciences (CASS) at Tartu University.

A *teacher-rated four-item measure* is used to assess classroom behavior. The measure originates from the Organization for Economic Co-operation and Development (OECD) Teaching and Learning International Survey (TALIS) 2013 teacher's questionnaire and asks to indicate how strongly a teacher agrees or disagrees with statements regarding classroom behavior on a four-point scale from (1) not at all to (4) completely agree [43]. There is no information available about the measures' validity and reliability.

Socio-demographic data is additionally collected from parents, including child gender and age, parental gender and age, parental nationality and home language, family structure, number of children, count of household members, financial situation of the household, current employment status and parental education. Teachers provide information about their gender and age, years of experience teaching and whether and what methods they are using in the classroom to support children's mental health.

#### 2.9.3 Data collection procedure

Table 1 describes the schedule of enrolment, intervention and assessments. Baseline information was collected at the beginning of the 1st academic year (3 weeks during September/October 2016), all participants were blind to allocation at this point. Post-tests will follow at the end of the 1st academic year (May 2017) and the 2nd academic year (May 2018).

**Table 1:** Schedule of data collection.

Time point	Study period			
	Enrolment	Evaluation		
	Pre-baseline	Baseline (October 2016)	First post-test (May 2017)	Second post-test (May 2018)
**Enrolment**				
Eligibility screen	X			
Informed consent	X			
Matching	X			
**Interventions**				
PAX GBG, intervention group				
Usual practice, control group				
**Parent-rated Assessments**				
Sociodemographic variables		X	X	X
Child's mental health and prosocial skills (SDQ)		X	X	X
Child's symptoms of attention deficit hyperactivity disorder *(*SNAP-IV*)*		X	X	X
**Teacher-rated assessments**				
Background variables		X	X	X
Child's mental health and prosocial skills (SDQ)		X	X	X
Teacher's sense of efficacy (TSES)		X	X	X
Classroom behavior		X	X	X
**Child-completed assessments**				
Response inhibition (Go/No-Go task)		X	X	

During the 1st year the teachers from intervention classes receive training as well as regular mentoring hence the first post-test demonstrates the effects following active implementation. Previous trials have showed that the immediate effect of GBG will be measurable by the end of 1st year, while follow-ups have found continuing positive effects of the intervention even if the intervention is not implemented after first year [54]. There is no cut-off for intervention during the study period, but as the teachers do not receive regular implementation support during 2nd academic year, the implementation of the intervention may decline during that time. The rationale for second post-test is to find out if the intervention has long-term effects, which will be measurable regardless if teacher's use or don't use the intervention.

First post-test is given priority in determining the intervention effects. The control group will not receive the intervention until the second post-testing in May 2018 has been completed.

Teacher's fill out SDQs for each child taking part in the study and the teacher's questionnaire (containing teacher's background information, TSES and the classroom behavior measure) during all three assessments. Paper versions of the questionnaires are sent to schools by NIHD.

Parents fill out electronic or paper versions (according to their preference) of parental questionnaires (containing sociodemographic, SDQ and SNAP-IV data) also three times. Electronic parental questionnaires are sent to parents via email. Paper versions are sent through schools and parents return filled out questionnaires to the NIHD in prepaid envelopes.

Subgroups of children complete computerized Go/No-Go tasks twice - in October 2016 and May 2017.

#### 2.9.4 Assessment of implementation

Intervention fidelity and children's exposure to the intervention are measured via (1) structured observations and (2) teachers' self-reporting.

1) Intervention adherence is evaluated via observations. Observations are carried out by mentors and by independent researchers during the 2016/17 school year.
  a) All independent researchers have passed the PAX GBG teacher's training and 2-h instruction. They visit one lesson per class during spring semester (February 2017) in first year. Researchers' data collection point was set for mid-term to assess intervention adherence after training is completed and teachers should be able to implement intervention correctly. All researchers conduct the first observation in pairs with a senior member of the research team to ensure the inter-rater reliability. Observers stay in the classroom for one lesson and ask the teachers to play a five-to 15-min PAX GBG game. They fill out the PAX GBG implementation rubric.
  b) Data collected from independent researchers will supplement information collected from mentors, who visit and observe classrooms regularly during the 1st academic year. As mentors are supporting the delivery of the intervention, additional data collected from independent researchers strengthens the research credibility. Mentors fill out two scoring rubrics (adapted from the PAX GBG implementation rubric and PAX GBG progress evaluation form) during each visit to the class.
  *PAX implementation rubric* contains 32 statements divided to seven dimensions: preparing students for the game (6 items), using the timer (3 items), team structure (4 items), recording student behavior (4 items), responding neutrally to misbehavior (4 items), game review after the game (4 items) and rewarding the students (7 items). Observers mark ‘no’ (0) or ‘yes’ (1) answers when the teacher used or did not use the method. Open-field text boxes are included to allow observers to record reasons for their ratings. If a teacher is using the methods correctly and scoring more ‘yes’ answers than ‘no’, then the intervention is implemented with higher fidelity.
  The *PAX progress evaluation form* helps mentors evaluate the overall development and use of the PAX GBG methods within the classroom. It features six dimensions: usage of the PAX GBG vision, vocabulary, cues, Tootle notes, additional kernels and a teacher's general attitude and openness towards the PAX GBG. In the beginning, a mentor has to give responses to statements about whether the teacher is using the method (‘true’ or ‘false’ answers) for each dimension. The next statements focus on the use of practices, for which the mentor has to choose between answers on a three-point scale (2 = ‘certainly true’, 1 = ’somewhat true or 0 = ‘not true’).
2) Children's exposure to the intervention is measured via teachers' self-reporting. Teachers use a web-based recording system to submit the number of games they have played during each week and the duration of each game played. This data is summed up across the school year and calculated into total number of games implemented and total number of minutes for which the PAX GBG was implemented.

### 2.10 Statistical analysis plan

Demographic and baseline characteristics at the pupil level will be summarized using means and standard deviations (or medians and inter-quartile ranges) for continuous variables and percentages for categorical characteristics.

For analyzing the intervention effects on child outcomes, two scenarios are presented. First one will be used when matching has been effective (correlation between paired cluster response means (SDQ total difficulties) at baseline is at least moderate i.e. above 0.4) and takes into account the matched-pair design. Multilevel linear models with three levels (child, school, and pair) will be used with continuous responses (i.e. SDQ total difficulties mean score at post-test), where intervention group (PAX GBG vs. control) will be entered at the school level as a fixed effect, random effects will model differences between pairs and variation of the intervention effect among pairs. Covariates (i.e. gender and pre-intervention score) will be included at child-level. Binary outcomes (i.e. SDQ bandings no risk/at risk) will be compared between trial arms using multilevel generalized linear models. Even though pair-matched cluster randomized trials have analytical limitations, the focus of analysis will be on the overall effect of intervention, which can still be estimated [55].

Second scenario will be used when matching has been ineffective (i.e. correlation is small). As suggested by research [56], [57], breaking pair matches and treating the design as completely randomized might be useful strategy. Two-level hierarchical linear models (child, school) will be used with continuous responses and Generalized Estimating Equations (GEE) with the ‘sandwich estimator’ for robustness for binary responses. This approach will help to find out if and how many children will transit from ‘at risk’ to ‘no risk’ banding.

Multilevel models will use maximum likelihood estimation to handle missing data, assuming that data is missing at random [58]. GEE requires stronger assumption of missing data mechanism and assumes data is missing completely at random.

Secondary analysis will seek to find out whether the intervention has effect on child's inhibitory control and on symptoms of ADHD. Differences from baseline to post-assessment between the two trial arms will be estimated using multilevel models, including child-level covariates (i.e. baseline response inhibition/baseline ADHD score and gender). Analyzing the intervention effects on teachers will be conducted with same scenario as the effects on children. Two level models will be used to take into account the pair-matched randomization and linear regression when matching has not been effective. Teacher's gender, age and years of experience teaching will be added as teacher-level covariates.

Explanatory analysis will be conducted to examine potential moderators of intervention effects. The aim is to find out if the intervention has different effect to children based on their baseline risk-status (i.e. no risk vs. at risk on SDQ) and socio-demographic factors (i.e. family structure and socioeconomic status), using four-level (with pair-matched design) or three-level (when matching was ineffective) models with repeated measures entered as first level variables. Secondary analysis about the effects to child's inhibitory control and to teacher's self-efficacy as well as explanatory analyses are purely exploratory, with any significant findings needing to be replicated in other studies as the power of these analyses is low.

Intervention fidelity will be reported descriptively.

Analysis will be made without multiplicity adjustments as the main purpose is to compare intervention and control group using a single primary variable. Secondary objectives are adding to the value of the intervention and supporting the hypothesis relating to the primary variable (positive effect on children's mental health).

Impacts are estimated on the basis of modified intention to treat (mITT) [59], excluding randomized schools deemed ineligible immediately after randomization. Even though this type of estimation can introduce bias [60], it is believed that excluding two randomized schools as well as their matched participants from the analysis is sensible. Thus, data is collected from 42 schools and analysis will be based on their input.

Trial reporting will follow the CONSORT guidelines [61].

## 3 Discussion

In recent years several prevention programs have been adapted and implemented in Estonian schools, such as the alcohol-prevention program, ‘Efekt’, anti-bullying programs, ‘KiVa’ and ‘Bullying-free school’, and mental disorder-prevention program, ‘PAX GBG’. At the same time, the evaluation of the effectiveness of interventions is deficient in Estonia [62], and this study is among the first to evaluate the intervention effects by conducting a randomized controlled trial (RCT).

This research helps to raise awareness of the importance of conducting impact evaluations and assessing the intervention's influence on children's well-being. In Estonia, the term ‘evidence-based’ is not formally defined by national authorities, which has resulted in many approaches and programs defining themselves as ‘evidence-based’. There is also confusion surrounding what kind of studies provide reliable evidence of a program's effectiveness. By carrying out an experimental study, an important step is taken towards improving the quality of studies and raising the standards of required evidence to prove that a program is effective in Estonia.

Although there is sufficient evidence for the effectiveness of GBG in general, and recent data has demonstrated the positive impact of the PAX GBG on children's mental health, this research serves as an opportunity to determine whether the PAX GBG functions in real-life conditions after adaptation to a new language and environment. The PAX GBG could protect Estonian children from a range of emotional and behavioral disorders and through that, lead to cost savings in the longer term.

This study also intends to contribute to the understanding of the mechanisms through which the PAX GBG might affect children's behaviors, such as their ability to inhibit a response. It is assumed that the PAX GBG might provide children with the tools that they need for self-regulation and, as such, prevent mental health disorders and risky behaviors. However, the study doesn't carry out mediator analysis. As we will collect data from children, teachers and parents, we will strengthen such research's credibility and validity through data triangulation. Another strength of this study lies in using questionnaires as well as behavioral task, the latter being considered to be more objective and less biased compared to questionnaires.

### 3.1 Limitations

Several methodological challenges influenced the design of this trial. First, cluster-RCTs require large sample sizes of participants to be able to detect small or moderate effects between the intervention and control group. It was thought to be impossible from the start of this study to involve large amounts of schools in this trial and, therefore, sample size calculations were not performed. There are altogether 472 schools in Estonia providing primary education in Estonian, of which most are small in size with an average of 10 students per classroom. Before the start of this trial, 79 schools were already implementing the KiVa or PAX GBG programs. As a consequence, it was possible to invite 164 schools to the trial, from which one-third showed interest after receiving several invitations.

Even though the number of schools participating in the trial was not large, the input from this study is important to guide policy-related decisions in Estonia as well as encourage similar study designs in other countries regardless of the limitations and challenges that may arise.

Secondly, the preliminary aim was to use a simple-cluster randomized design. Based on the implementation-related goals, the final decision was to perform a matched-cluster randomization trial instead, which has its own weaknesses. It is unknown whether the matching was effective as the variables matched are not expected to be correlated with the outcomes. Hence, it is difficult to predict whether matching has actually improved the power of the study or not.

Another issue involves the impact estimations, which will be based on mITT. The initial plan was to conduct an ITT analysis, the gold standard for assessing the superiority of the intervention in randomized trials [63]. Unfortunately, it was not possible to keep two schools in the study and thus their matched schools were also excluded. At this moment it is assumed that this did not increase the potential for bias.

One explicit limitation of this study is its focus on outcomes rather than the factors influencing the outcomes. Prior evidence has shown that teacher-related factors, such as their personal resources and attitudes, are associated with PAX GBG implementation success [64]. It would be interesting to establish how the intervention aligns with Estonian teachers' approaches to teaching. Furthermore, it would be valuable to discern which individual, school-level and environmental factors influence the adaptation and implementation of the intervention. To enhance the implementation and mentoring, as well as to ensure sustainability, additional qualitative data collected from mentors, school administrators and teachers would be necessary. Those are all areas of further research that would be conducted later if there are sufficient resources available.

## 4 Conclusion

There is a strong need in Estonia, and in Europe, in general, to bolster research into mental health and well-being among children, including the outcomes of interventions [65]. Schools often implement preventive interventions to improve children's well-being, but it is frequently unknown if these activities achieve their objectives and make a positive difference. At the same time, several challenges hinder evaluating the effects of school-based interventions, such as the inability to recruit and retain large sample of schools. Nevertheless, impact evaluations are important to demonstrate causality as well as to find out whether adapted programs work, and if they do so, across different environments.

## Funding

The study is funded by the European Social Fund and Ministry of the Interior in Estonia.

## Competing interests

None of the authors have competing interests.

## Authors' contributions

KS was the lead author of this study protocol. KS, AT and KK designed the study. AS advised on statistical aspects. KA led the development of Go/No-Go task and provided expertise on psychological factors. MS and KT supplied methodological guidance throughout the design of the study. All authors contributed to the content of the study protocol, read and approved the final manuscript.
